# Implementation strategies for the introduction of the RTS,S/AS01 (RTS,S) malaria vaccine in countries with areas of highly seasonal transmission: workshop meeting report

**DOI:** 10.1186/s12936-023-04657-5

**Published:** 2023-08-23

**Authors:** Corinne S. Merle, RTSS-SMC working group, Ndeye A. Badiane, Cyriaque Dossou Affoukou, Souliatou Yolande Affo, Salako Luc Djogbenou, Aurore Hounto, Sidzabda Christian Bernard Kompaore, Issa Ouedraogo, Marcellin Joel Ateba, Andreas Ateke Njoh, Dominique Bomba, Shalom Ndoula Tchokfe, Innocent Mbulli Ali, Fatou Camara, Momar Mbodj, Stephen Sosler, Josea Rono, Lizzie Noonan, Muniratu Venu, Mohamed Naziru Tanko, Thomas Gyan, Margaret Gyapong, Jose Ernesto Nante, Humberto Imbunda Intchala, Mohamed Binné Camara, Gassim Cisse, Jonas Emile Loua, Rose Jalang’o, Paul Milligan, Susana Scott, Kevin Baker, John Sande, Brenda Lupafya Mhone, Aissata Kone, Yacouba Coulibaly, Ibrahima Diarra, Andre Marie Tchouatieu, Miriam Aliaprieto, Ibrahim G. Diallo, Yahaya Abou, Abdoul Nasser Assan, Hadiza Jibril, Nigeria Nmep, Nelson Chibueze Eze, Garba Bello Bakunawa, Emmanuel Shekarau, Jamilu Nikau, Scott Gordon, Elizabeth Ann Wilskie, John Bawa, Ndeye Astou Badiane, Katharine Sturm-Ramirez, Jean Pierre Kidwang, Doudou Sene, Jean Standeur Nabykaly, K. A. Abdoulaye, Mahamat Saleh Issakha Diar, Abdraman Mahamat Addi, Branwen J. Hennig, Gildas A. Yahouedo, Olimatou Kolley, Sidat Fofana, Mbye Nijetinah Atcha-Oubou, Hezouwe Looky Djobo, Rokhaya Mbaye, S. Y. Ousmane, Jean Louis Abdourahim Ndiaye, S. E. C. Amadou, Ibrahima Mariétou Mbaye, Fatimata Bintou Sall, Ndèye Fatoudiop, Léontine Ndogou Bakhoum, El Hadji M. B. Diouf, L. Y. Almamy Youssouph, Khady Ndiaye, Belynda Amankwa, Les Ong, Cecilia Oh, Bill Wirngo, Liang Kung, Judith Hedje, Lydia Tuitai, Oniovo Efe-Aluta, Mgaywa Magafu, Spes Ntabangana, Mouctar Kande, Rafiq Okine, Mary Hamel, Jenny Walldorf, Eliane Furrer, Kristen Kelleher, W. U. Lindsey, Mayuko Takamiya, Cynthia Bergstrom

**Affiliations:** Special Programme for Research and Training in Tropical Diseases (TDR), World Health Organization, Avenue Appia 20, 1211 Geneva 27, Switzerland

**Keywords:** Malaria transmission, RTS,S vaccine, EPI, Seasonal malaria chemoprevention (SMC), Implementation research, Low- and middle-income countries (LMIC)

## Abstract

A workshop on implementation strategies for the introduction of the RTS,S/AS01 (RTS,S) malaria vaccine in countries with areas of highly seasonal transmission, was held as a hybrid meeting in Dakar, Senegal, and online, 23–25 January 2023. Delegates from Expanded Programmes on Immunization (EPI) and National Malaria Control Programmes (NMCPs) from 13 African countries, and representatives from key stakeholders participated. RTS,S is the first malaria vaccine to be recommended by the World Health Organization (WHO). The recommendation followed pilot implementation of the vaccine in Ghana, Kenya and Malawi, which showed that introduction of the vaccine was highly effective at scale, and was associated with a 30% reduction in hospital admissions with severe malaria in age groups eligible to have received the vaccine and no evidence of the safety signals that had been observed in the phase 3 trial. Clinical trials in Mali and Burkina Faso, showed that in children receiving Seasonal Malaria Chemoprevention (SMC), providing the vaccine just prior to high transmission seasons, matching the period of highest efficacy to the peak transmission season, resulted in substantial reduction in the incidence of clinical malaria and of severe malaria. While SMC has been successfully scaled-up despite the challenges of delivery, there is no established platform for seasonal vaccine delivery and no real-world experience. The objectives of this workshop were, therefore, to share experiences from countries that have introduced the RTS,S vaccine in routine child vaccination programmes, with SMC-implementing countries as they consider malaria vaccine introduction, and to explore implementation strategies in countries with seasonal transmission and where EPI coverage may be low especially in the second year of life. Practical implementation challenges, lessons learned for vaccine introduction, and research questions, towards facilitating the introduction of the RTS,S (and other malaria vaccines) in countries with seasonal malaria transmission were discussed.

**Supplementary Information:**

The online version contains supplementary material available at 10.1186/s12936-023-04657-5.

## Background

In October 2021, the World Health Organization (WHO) recommended the use of RTS,S/AS01 (RTS,S) malaria vaccine for the prevention of *Plasmodium falciparum* malaria in children living in regions with moderate to high malaria transmission. The recommendation of this first malaria vaccine was based on findings from the pilot introduction and evaluation of the vaccine in Ghana, Kenya and Malawi (launched in 2019 and ongoing through 2023), and other RTS,S research evidence showing that the vaccine can be delivered effectively, has a strong safety profile and can have a significant impact in real-life childhood vaccination settings. High uptake of the vaccine was achieved in the pilot countries, showing strong community demand and acceptance of the vaccine by health workers and communities.

The WHO recommends the RTS,S malaria vaccine be provided in a schedule of 4 doses to children from 5 months of age. For countries with areas of highly seasonal transmission of malaria, the WHO recommendation recognized the impact of aligning the administration of the vaccine just prior to the malaria season, and includes an optional alternative 5-dose seasonal delivery strategy to optimize vaccine efficacy, as well as the additional impact of coordinating the provision of the vaccine with malaria chemoprevention (SMC). Because there is no real-world experience in delivering the malaria vaccine using the seasonal strategy, countries that choose to adopt such a strategy are “strongly encouraged to document their experience, including the vaccine effectiveness, feasibility and occurrence of any adverse events, to feed into future guidance updates” and in addition WHO encouraged “international and national funders to support relevant learning agendas” [1].

This workshop was convened by the OPT-SMC project in collaboration with The Access & Delivery Partnership (ADP) partners, the WHO Malaria Vaccine Implementation Programme (MVIP) of the Department of Immunization and, the Vaccines and Biologicals and the WHO regional office for Africa. The OPT-SMC project supports 14 countries in West and Central Africa to conduct implementation research for optimizing the effectiveness of SMC, working in partnership with the University of Thiès in Senegal, the Special Programme for Research and Training in Tropical Diseases (TDR), Medicines for Malaria Venture (MMV), and the London School of Hygiene and Tropical Medicine (LSHTM), with funding from the European and Developing Countries Clinical Trials Partnership (EDCTP). The ADP works with low- and middle-income countries to ensure life-saving medicines and health technologies reach the people who need them. This partnership is led by the United Nations Development programme (UNDP), in collaboration with PATH, the WHO Regulatory Department and TDR, supported by the Government of Japan.

The aim of the workshop was to bring together representatives from Expanded Programme on Immunization (EPI) and National Malaria Control/Elimination Programmes (NMCP/NMEP) in countries with highly seasonal malaria, and relevant stakeholders, to share experiences and lessons learned during pilot implementations, to consider vaccine scheduling and delivery in the context of seasonal malaria transmission and varying levels of EPI coverage. The workshop aimed to support planning of introduction of the vaccine to optimize its impact in reducing child morbidity and mortality caused by malaria.

## Workshop objectives

- To review the current evidence on the RTS,S malaria vaccine in terms of evidence on vaccine efficacy and impact, and safety
- To share experiences across countries that have introduced the RTS,S vaccine in routine child vaccination services in Ghana, Kenya, Malawi, and SMC-implementing countries that are considering malaria vaccine introduction, to better understand the practical implementation challenges and lessons learned for vaccine introduction
- To discuss regulatory and supply management issues when introducing the RTS,S vaccine in the health systems
- To discuss implementation strategies and mode of delivery in countries with seasonal transmission and low or moderate EPI coverage during the 1st and 2nd year of life
- To discuss the operational/implementation research needs to document the implementation of RTS,S in terms of uptake and effectiveness, safety and acceptability.

**Fig. 1:**
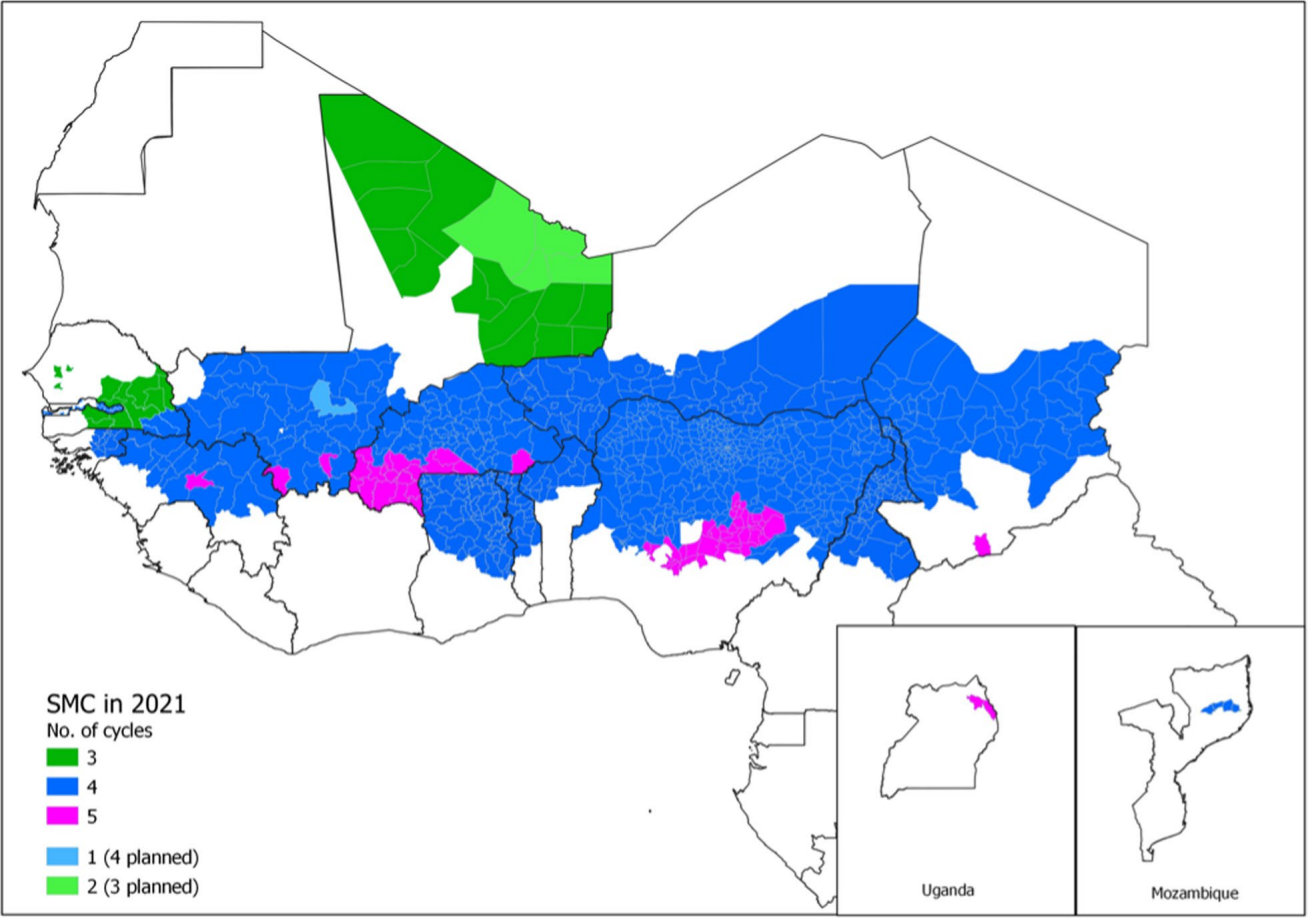
Areas which implement seasonal malaria chemoprevention (SMC), courtesy of OPT-SMC project

The participants were representatives from the National Malaria Control/Elimination Programmes (NMCP/NMEP) and Expanded Programmes of Immunization (EPI) from 13 countries in West and Central Africa that are currently implementing SMC (see Fig. 1). EPI and NMCP representatives from Kenya, Malawi and Ghana, where the RTS,S vaccine has been piloted were also present [2]. Key stakeholders involved in vaccine implementation and or malaria control strategies included WHO Department of Immunizations, Vaccines and Biologicals, WHO Global Malaria Programme, WHO regional office for Africa, WHO country office Senegal, TDR, Gavi (the Vaccine Alliance), Medicines for Malaria Venture (MMV), United Nations Development Programme (UNDP), PATH, and academics from University of Thiès, London School of Hygiene & Tropical Medicine (LSHTM), Malaria Consortium and others (see RTSS-SMC working group details). For the workshop agenda and presentations see additional files 2 and 3. Workshop recommendations are summarized in the conclusion section and implementation research questions in Additional file 1.

## Day 1 scene-setting

The workshop was opened by the chancellor of University of Thiès (Senegal), Prof Ramatoulaye Diagne Mbengue, followed by welcome remarks from the meeting co-organisers Prof Jean-Louis NDiaye (University of Thiès, Senegal) and Dr Corinne Merle (TDR, WHO, Switzerland).

Brief overviews of the OPT-SMC project (presented by Ndiaye) and the ADP initiative (presented by Cecilia Oh, UNDP Bangkok, Thailand) also highlighted the benefits of promoting inter-country collaboration, and sharing of information and expertise, particularly with respect to implementation research (IR) activities, within their respective networks. These benefits also aligned with the premise of this workshop to facilitate the successful introduction of the RTS,S vaccine in countries with different malaria prevalence and EPI coverage and given the limited availability of vaccine.

*RTS, S malaria vaccine current evidence, including efficacy, safety, feasibility and impact and update on the WHO review of the R21/MatrixM vaccine* (Speaker: Mary Hamel, Malaria Vaccines, WHO, Geneva, Switzerland).

The global trend in malaria morbidity and mortality remains high, with 241 million cases and 627,000 deaths reported in 2020, with over 80% of the burden of malaria occurring in children in Africa. In March 2022, the WHO released a position paper on the use of the RTS,S/A01 malaria vaccine for the prevention of *P. falciparum* malaria in children living in regions with moderate to high transmission as defined by the WHO [1]. The paper included the recommendation that the vaccine be provided in a four-dose schedule in children from five months of age in the context of comprehensive national malaria control plans. Additionally, countries may consider providing the RTS,S vaccine seasonally, to maximize impact by timing vaccination to just before the period of highest malaria transmission. The recommendation also includes flexibility to allow for countries to schedule the vaccine to optimize delivery, for example, to align 4th dose with other vaccines or health interventions in second year of life.

Pilot implementations to understand the vaccine in routine use will continue through 2023 in Malawi, Ghana and Kenya. These have shown that the vaccine introduction is feasible, safe, impactful and equitable. Importantly, adding a malaria vaccine to current interventions, e.g. insecticide-treated bed nets (ITN), increases access to malaria preventive tools. As of December 2022, 3.8 million vaccine doses have been administered with 1.2 million children receiving at least one dose. In all three countries vaccine coverage is good for the first three doses (> 73%), but drops for the fourth dose (54–72% of children who received the 3rd vaccine dose received the 4th dose).

Modelling predictions indicate a significant public health impact across a wide range of malaria transmission settings, and a high level of cost effectiveness at a cost 10 USD per dose with an estimated 400 deaths averted per 100,000 fully vaccinated children.

Demand for the RTS,S vaccine is very high, outstripping currently available supply. A number of other malaria vaccine candidates are in clinical development, including R21/Matrix-M. This vaccine is currently in phase III trials, and initial (unpublished) and phase II trial data [3] show high efficacy, similar to that observed with RTS,S/AS01, when provided seasonally [4]. The WHO is in the process of reviewing the efficacy, safety, and programmatic suitability of R21/MatrixM. If recommended for use, it could be important means to increase vaccine supply to meet demand.

*Vaccine efficacy and seasonality of malaria* (Speaker: Paul Milligan, LSHTM, London, UK).

The WHO estimates that 12 countries in West and Central Africa with areas of highly seasonal transmission account for 50% of the worldwide total of malaria deaths each year [5]. Optimal implementation of the malaria vaccine in these countries is, therefore, a priority. Seasonal malaria chemoprevention (SMC) has been scaled-up effectively despite the challenges of delivery, frequencies of drug-resistant parasite genotypes are low, and treatments remain highly effective [6], but SMC does not provide complete protection and additional measures are needed. Trials in Mali and Burkina Faso in children receiving SMC, showed that 5 doses of RTS,S (3 primary doses and two annual boosters) administered seasonally reduced the incidence of clinical malaria over 3 years by 63% and severe malaria by 71%, in addition to the protection provided from SMC [4]. Taking into account the protection that SMC and long-lasting insecticidal nets (LLINs) provide, a very high level of personal protection (more than 90%) is now potentially possible through the combined use of LLINs, SMC and the malaria vaccine, but there are significant implementation challenges. RTS,S is not recommended before 5 months of age, but children should start their course of vaccination as soon as possible from this age. Because protection from the vaccine is greatest in the few months after vaccination, it is desirable for booster doses to be timed just before the high malaria season starts for the maximum benefit. Strategies being considered involve providing the primary three doses and the fourth dose according to age, or a ‘hybrid’ strategy with the three primary doses given a month apart starting as soon as possible from 5 months of age, and a 4th dose just before the start of the next malaria season or, if by the start of the season the child has only just received their third dose, just before the following season, and a 5th dose a year after the 4th. As uptake of vaccines tends to be lower in the second year of life, novel strategies will be needed to reach children with booster doses. The ‘hybrid’ schedule, with seasonal booster doses administered through campaigns or seasonal intensification of vaccine delivery with strengthened outreach could provide a means of strengthening vaccine delivery in the second year of life, and a platform for delivering a 5th dose. Selection of the most suitable options for vaccine implementation needs careful consideration, taking into account the local epidemiology, vaccine efficacy trajectories, current level of vaccine uptake in the second year of life, and availability of vaccine doses.

*Update on supply of RTS,S with allocation framework* (Speaker: Eliane Furrer, Malaria Vaccines, WHO, Geneva, Switzerland).

This presentation covered the current malaria vaccine supply situation and the Framework for allocation of limited vaccine doses. Initial vaccine supply is expected to be insufficient to meet the high demand from malaria-endemic countries. Given that over 28 countries have already expressed interest in introducing the vaccine, the current discrepancy between demand and supply is vast (80 to 100 million doses needed per year vs 18 million doses available between 2023 and 2025).

The WHO was tasked to develop a framework to guide in a transparent, principles-and evidence-based manner how initial limited malaria vaccine doses should be allocated [7]. The Framework, which was endorsed in July 2022, will be applied following each Gavi application round to all successful proposals, i.e. proposals recommended for approval by Gavi’s Independent Review Committee. The framework covers governance principles (transparency, inclusiveness, accountability), ethical principles for allocation (greatest need, maximum health impact, equity, fair benefit sharing), and additional key considerations (including commitments to MVIP areas, continuity/sustainability of access, minimization of wastage and delayed use of doses) that will be employed for prioritization of the vaccine allocation. The first priority principle is to allocate the vaccine to areas of greatest need, that is, areas where the malaria disease burden in children and the risk of death are highest. The need will be assessed through a proxy measure based on a composite index combining sub-national measures of malaria burden (P. falciparum parasite prevalence or incidence rates in children) and under-five all-cause mortality. There will further be a ‘solidarity cap’ (currently set at 1 million doses per country per year) to enable a larger number of countries to access the vaccine for initial roll-out in greatest need areas. If the cumulative vaccine dose requirements for greatest need areas in approved countries cannot be met, the second priority principle (i.e. to maximize health impact) will be applied to further prioritize across countries, using a proxy measure of the drop-out”rate” between the third dose of Diphtheria-tetanus- pertussis vaccine (DTP3) and the first dose of measles-virus containing vaccine (MCV1).

Whilst the framework clarifies the allocation principles and criteria upfront, it does not remove all uncertainties, e.g. the timing of a country’s application to Gavi, the number of approved countries and their needs, and the dynamic nature of the supply situation all affect when countries may be able to access vaccine doses. The key implications of the current situation are that not all interested countries will have access to the vaccine initially, and that those who receive a supply allocation, will have to roll-out the vaccine in a phased approach, starting in areas with greatest need.

To help manage expectations and support decision-making and planning, transparency and communication will be maintained regarding supply availability over time and allocation decisions.

*Gavi malaria vaccine programme update* (Speaker: Stephen Sosler, Gavi, Geneva, Switzerland).

Gavi is in its 5th phase of accelerating access to 17 vaccines globally. In late 2021 Gavi approved funding for the RTS,S malaria vaccine through a recommendation for a new Malaria Vaccine programme. A coordinating body for this programme is co-chaired by the WHO and Gavi, and includes other organizations joining a stakeholder group (i.e. PATH, Global Fund, UNICEF, World Bank), to ensure coordination across immunization and malaria partners.

Gavi support for the RTS,S malaria vaccine comprises i) facilitation of the phased, sub-national introduction of the vaccine into national vaccination schedules in areas with moderate to high P. falciparum malaria transmission as defined by the WHO; ii) procurement of vaccine doses and associated supplies; and iii) the provision of technical support to countries towards the development of applications and the implementation of the malaria vaccine programme. Notably, Gavi currently does not provide support for malaria vaccine campaigns or catch-up vaccination.

There are four Gavi application windows per year with guidelines for support, application process and vaccine funding available in French and English, via the Gavi website [8]. The first Gavi application round has seen unprecedented demand with 13 applications in early 2023. This indicates further that there is an overarching need to close vaccination coverage gaps exasperated by Covid-19.

The initial cost of the vaccine is approximately $38/child/4 doses. To facilitate affordability and country uptake of the vaccine, Gavi approved an exceptional, time-limited co-financing approach with initial self-financing ($0.20/dose), followed by a preparatory transition phase and an accelerated transition ramping up co-financing from 20 to 100% of the vaccine price over eight years.

A Gavi-led market shaping roadmap has also been developed and aims to increase supply, reduce the price, materialize sustainable demand and incentivize product innovation for next generation vaccines. Meanwhile all available vaccine supply for 2023–25 and its procurement was contracted via UNICEF.

*Regulatory consideration for introduction of RTS, S in countries with seasonal malaria* (Speaker: Lydia Tuitai, The WHO regional office for Africa, Kenya).

The WHO regional office for Africa has led efforts towards regulatory considerations through a joint review of RTS,S. A team of experts from marketing authorization, pharmacovigilance, clinical trial authorization with representation from 17 countries was convened under the African Vaccine Regulatory Forum (AVAREF) with support from the European Medicines Agency (EMA) at WHO. This resulted in non-binding recommendations endorsement in mid-2022. These put in place mechanisms for regulatory pathways for registration of vaccines, and timelines after vaccine prequalification though an agreed action plan. Importantly, national regulatory agencies (NRAs) can use the non-binding recommendations to inform their decision without undertaking a full review themselves. This is facilitated by applicants submitting dossiers whilst NRAs are given access to the WHO prequalification assessment report, EMA opinion report and other relevant data.

To date the three countries that piloted introduction of RTS,S – Ghana, Kenya and Malawi – all have their dossier under finalization locally with targeted submission to the respective NRA in early 2023.

*Overview of SMC (including target population, eligible geographic area, model of delivery, integration with other preventive measures)* (Speaker: André Tchouatieu, Medicines for Malaria Venture, Geneva, Switzerland).

Seasonal malaria chemoprevention (SMC) is recommended in areas with high malaria transmission, i.e. where at least 60% of yearly malaria cases are concentrated within a 4 month period each year, usually coinciding with the rainy season. SMC is the intermittent administration of a curative dose of anti-malarial medicine during the malaria season, regardless of the infection status of a child. Its aim is to establish anti-malarial drug concentrations in the blood to clear existing and prevent new infections at the height of the malaria season.

Recommendations regarding the geography and transmission intensity, number of doses or cycles, specific drugs recommended and taking into account age-based risk among children were recently updated and published [9]. These allow for greater flexibility to adapt to epidemiological settings, whilst increasing safety and efficacy of the intervention.

SMC administration is not trivial, given the need for 3–5 months of delivery through door-to-door campaigns using community health care workers and/or volunteers. Directly observed therapy (DOT) and/or coupling of SMC with other public health interventions can significantly increase effectiveness, however sustainability of such approaches may be limited due to associated costs. To date around 45 million children have been covered by SMC across Africa, with steady increase in delivery despite Covid-19, and an exponentially higher coverage in Nigeria.

*Lessons learned and practical experience from pilot introduction of the RTS, S malaria vaccine: implementation in routine child immunization programmes and possible implications for implementation through SMC or other mass drug administration programmes* (Speakers: John Sande, EPI, Malawi; Brenda Lupafya Mhone, EPI, Malawi; Rose Jalang’o, EPI, Kenya; Naziru Tanko Mohamed, EPI, Ghana; and Muniratu Venu NMCP, Ghana).

**Fig. 2:**
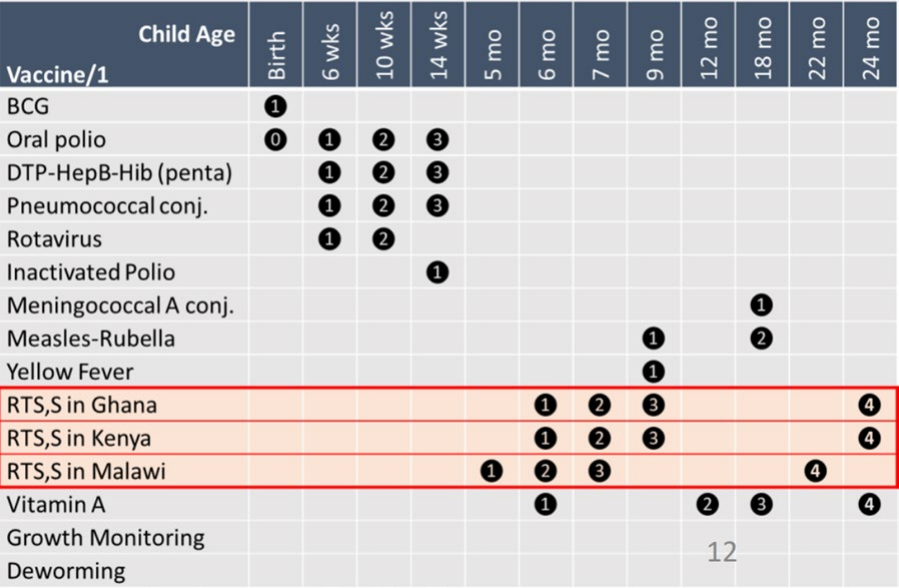
EPI vaccine schedules in RTS,S pilot countries Ghana, Kenya and Malawi (with thanks to WHO)

Malawi, Kenya and Ghana were the first countries to introduce the RTS,S malaria vaccine through routine immunization programmes in 2019 as part of the WHO-coordinated Malaria Vaccine Implementation Programme. Representatives from the EPI and/or NMCPs gave an overview in each of their settings, focusing on lessons learned to date. In each country doses 1–3 were administered at slightly different time points in the first year of life, with the fourth dose at around two years of age (see Fig. 2).

### *Malawi*

- Ministry of Health commitment at highest levels facilitated successful programme implementation, with planning and coordination at national level involving all key stakeholders including EPI and NMCP
- Social mobilization and community engagement was critical for vaccine demand creation amongst caregivers, key opinion leaders, local and religious leaders. Enhanced social mobilization for the 4th dose of the vaccine is needed and ongoing.
- Supportive supervision (technical support in communities) to identify challenges and provide timely solutions was essential, particularly with respect to health care workers addressing caregivers’ questions and relaying relevant health messages consistently.
- Stakeholder engagement at district level facilitated reciprocal learning
- Post-introduction evaluation is essential to assess the benefits and caveats of introduction of a new vaccine into routine immunization

### *Kenya*

- Challenges observed included a low fourth dose coverage, health care worker challenges with understanding eligibility criteria for vaccine administration, and insufficient investment for effective social mobilization.
- Active community engagement was seen as central to addressing these challenges, as was the coordination between EPI and NMCP teams, national and subnational stakeholder engagement, integration of the malaria vaccine into the broader health system and sustained advocacy for second year of life platform vaccines.
- Opportunities for EPI/NMCP programmatic coordination was seen to be particularly beneficial with respect to maximising data driven decision making; advocacy for continued investment on malaria; communication and community engagement; and integrated service delivery.

### *Ghana*

- Good collaboration between the National Malaria Elimination Programme (NMEP), EPI and national regulatory agency (NRA) is essential for the operationalization of plans
- The vaccine introduction plan should address health systems strengthening and integration with other child health interventions across the life course
- Healthcare workers must be adequately educated to ensure effective communication with caregivers, timing training adequately
- Existing defaulter tracing systems must be reviewed and strengthened to reduce drop-out rates
- Catch-up campaigns may be necessary to optimize the uptake of the 4th dose, including leveraging the second year of life strategies
- Appropriate strategies should be developed to reach eligible children in difficult to reach populations (island/riverine communities, urban and peri-urban areas, mobile populations)
- Where available, the use of electronic vaccination registries can support improving data quality and defaulter tracking
- Strengthen vaccine quantification and forecasting at all levels to prevent avoidable vaccine stock-outs
- The systems for surveillance of adverse event of special interest (AESI) were poorly understood by health care providers and led to low reporting, management will require careful consideration going forward.

All three countries reported the beneficial value of the integration of EPI and NMCP activities. However, there were challenges of achieving high coverage for the fourth dose of the RTS,S vaccine within the routine vaccination programmes. This finding is consistent with lower coverage of other EPI vaccines given in the second year of life. The key going forward will be identifying how to tackle the reduction in coverage for older children.

*Qualitative findings and lessons learned from the pilot countries: perceptions of malaria, the vaccine and other interventions* (Speaker: Scott Gordon, PATH, Seattle, USA).

Facilitators and barriers to the uptake of the RTS,S vaccine were explored through qualitative studies in the three pilot countries. The findings across all three countries and settings indicated a trajectory of growing trust in the vaccine over time, driven by foundational trust in health systems and vaccines, and increasing with RTS,S-specific trust with growing understanding of the benefits and safety aspects of the vaccine. Initial hesitations about RTS,S were overcome with clear communication though trusted channels within the health system, coupled with additional facilitators such as encouragement from within the wider community (e.g. family members). Importantly, by the time the third dose of the vaccine was administered, caregivers understood that RTS,S was partially protective and that therefore the continued practice of other prevention measures and professional care seeking in the event of fever, is essential. Data further showed consistent bed net use over time as RTS,S was introduced. Similarly, prompt treatment seeking for fever or suspected malaria continued for children receiving the vaccine.

Barriers to uptake and adherence were identified and differences in these barriers were reviewed by dose uptake category. Uptake of dose 1 seems primarily driven by trust in the health system, with the very limited incidents of non-uptake due to refusals, stemming from low vaccine confidence, compounded by country-specific issues. Routine RTS,S promotion or information, education and communication strategies appeared to be insufficient to reach or persuade those limited populations most-at-risk of missing dose 1. The reasons for non-uptake of dose 2 or 3 (more common among respondents) are similar to those for non-uptake of dose 1. However, partial protection through the vaccine was not an impediment for uptake or use of other interventions.

Completion of the full four dose vaccination course was seen to be positively influenced by reminders of vaccine visits, encouragement by health care workers and increasing trust in RTS,S specifically. Routine use of the child health book to remember vaccination dates and vaccination visit reminders also contributed to uptake. Conversely, service interruptions and an abundance (rather than lack) of confidence in the vaccine associated with complacency were shown to contribute to dose 4 defaulting. Personal life barriers also appeared to play a role in the lack of uptake of dose 4. Overall, the findings are helpful to inform future strategies for the introduction of the vaccine in different settings.

*Practical considerations for RTS, S malaria vaccine supply chain and limited supply management at country level* (Speaker: Betsy Wilskie, PATH, Seattle, USA).

To stimulate the discussion of implementation of the malaria vaccine particularly in countries with seasonal transmission planned for day two of the workshop (and beyond), four key areas of considerations were flagged – each of these is associated with a significant amount of complexity. 1) Targeted distribution in the context of the need for some countries to prioritize areas of highest need given the limited vaccine supply. 2) Cold chain capacity and a need for analysis thereof across all levels of service delivery in individual countries to evaluate equipment and maintenance needs. 3) Wastage minimization based on accurate data on vaccine wastage to facilitate correct planning for vaccine procurement. And lastly, 4) flexible data driven supply plans to inform reverse logistics and re-distribution, staggered delivery schedules and cold chain equipment purchase and planning.

## Day 2 implementation strategies and mode of delivery for combined RTS, S and SMC programmes

### Round table on plans for RTS, S roll-out in countries who applied in January 2023 for RTS, S vaccine procurement

Short presentations were given by country representatives from Benin, Burkina Faso, Cameroon, Tchad and Niger, outlining at what stage each of these countries are at in terms of RTS,S vaccine introduction planning given WHO recommendations and Gavi requirements for accessing support.

A rich discussion ensued, with participation from both the audience in the room and online. Questions and answers fed into the group discussions on days 2 and the general discussion on day 3, and are therefore captured under the relevant sections in this report.

*Considerations and possible modalities for introduction of RTS,S malaria vaccine in countries with seasonal malaria* (Speaker: Rafiq Okine, WHO, Geneva, Switzerland).

The WHO recommendations for use of the RTS,S malaria vaccine allows for flexibility, for countries to adapt the schedule to their needs. Notable key findings from previous and ongoing studies were laid out: i) Protective efficacy is seen after completion of the primary series (3 doses), the 4th dose significantly prolongs the duration of protection; ii) schedule considerations represent a trade-off between optimizing vaccination coverage and providing the best protection during the period of greatest risk; and iii) reaching children aged two and beyond is difficult with drop-out rates increasing over time. Furthermore, epidemiological studies show that in areas of high perennial malaria transmission, a considerable risk (20–40%) of severe malaria and death remains beyond age 3. In highly seasonal transmission settings, this risk may still be substantial at least through age 5. Hence, countries may align the choice of schedules with the local disease epidemiology and risk-profile ensuring that majority of children are protected during the period when they are at the highest risk of severe disease and mortality.

These findings are particularly pertinent to settings with highly seasonal malaria transmission in relation to scheduling and delivery of the RTS,S malaria vaccine. Providing the primary series and additional annual doses prior to the high transmission season offers high protective efficacy.

Three main delivery models could therefore be considered, 1) age-based through routine EPI delivery platforms, 2) seasonally timed doses through vaccination campaign style delivery, or 3) a hybrid approach. Each of these allows for some flexibility and each is associated with pros and cons that need to be considered context specific (see Table 1). The context depends on the epidemiology or burden, vaccine efficacy and waning of protection over time, the likelihood of reaching high vaccine coverage and, therefore, impact, and programmatic implications including public engagement and costs. Seasonal malaria vaccination is a new area with many outstanding questions to answer on delivery strategies, optimal number of annual doses, vaccination schedule. Key questions that are as yet unanswered are (Table 2):

- What is the optimal interval between dose 3 and 4?
- What should the target age group for the campaign style delivery be?
- What is the safety and efficacy of a > 5-dose strategy?
- What is the best delivery strategy depending on the malaria transmission intensity and length of the transmission season?
- Can improved delivery be achieved through integration with other interventions?

**Table 1:** Pros and cons for different delivery strategies for RTS,S malaria vaccine

Delivery strategy	Pros	Cons
1.1. Age-based delivery through the routine EPI using 4-dose schedule	• Delivery through a well-known and established system and opportunity to strengthen RI • Caregivers and HCWs may be more familiar with this strategy • Comparatively less resource-intensive than a campaign style delivery	• High drop-out rate between doses • Relies on well-functioning EPI and health delivery system to achieve good coverages • Potential challenges with 4th dose coverage
1.2. Age-based delivery through the EPI using 4-dose schedule with timed/seasonal PIRIs/catch-up (before the high transmission season)	• Provides opportunity to catch up on other vaccines or interventions • Has the potential to reduce dropout rates with good 4th dose coverage compared to “regular” routine delivery	• Need for resources to sustain PIRIs • *other potential challenges as above*
2.0. Seasonally-timed doses through campaign style delivery	• Has the potential to reach high coverage for all doses • Leverages the period of high vaccine efficacy vs. high malaria transmission and thus provides greater protective efficacy • May be suitable for specific populations (difficult to reach with poor health service delivery)	• Resource intensive • May disrupt provision of other essential health services if not properly integrated and planned (vaccination campaigns and SMC)
3.1. Hybrid with primary 3 doses delivered through the routine EPI (age-based) with the annual doses (seasonal boost) given through a vaccination campaign before the high transmission season	• Primary doses delivered through an established platform • Timed annual doses before the high malaria transmission doses for children who have completed primary series provides greater protective efficacy	• Potential of poor coverage of primary doses if EPI delivery system is suboptimal • Vaccination campaign may require sustainable financing • Variable interval between 3rd and 4th dose vs. current guidance
3.2. Hybrid – with Primary 3 doses delivered through the routine EPI (age-based) with the annual doses (seasonal boost) given through a media campaign/intensified communication (using routine EPI delivery) before the high transmission season	• Advantages mainly same as above in addition to providing the opportunity to optimize delivery with other child health interventions through the media campaigns	• Need for sustainable funding to ensure effective community mobilization through media campaigns • May not be the best strategy for difficult to reach populations (will require extensive outreach services, if this planned to be HF-based) • May result in low coverages and drop-out if media campaigns are not effective

**Table 2:** Pros and cons for combined/integrated RTS,S malaria vaccine and SMC delivery

PROS	CONS
Very high protection by integration of malaria vaccine and SMC interventions	Different human resource, training and expertise needs for SMC and RTS,S (tablet vs injectable intervention)
Leverage on SMC intervention for social mobilization to increase vaccine uptake	Competing priorities, risk of favouring one intervention of the other, risk of frustration or even boycott for both SMC and RTS,S targets
Utilisation of SMC tracing mechanisms to overcome vaccine-drop out	Requires more resources, additional operational costs
Opportunity for integrated systems approach to public engagement	Complex logistics—question about feasibility of running multiple separate and overlapping campaigns
Positive experience of integration of BCG vaccination with SMC in Guinea	Concerns regarding the feasibility of adverse drug event monitoring
Positive experience of integration of EPI and NMCP in RTS,S pilot countries	Door-to-door vaccination strategy not appropriate for injectable vaccines

Okine reiterated that going forward WHO strongly recommends that countries document lessons learned from seasonal vaccination strategies, especially regarding operational feasibility, vaccine efficacy and safety.

### Group work on implementation strategies depending of model delivery

Based on evidence considered and discussion ensued throughout the workshop, there appeared to be consensus towards favouring a hybrid delivery mode as ‘ideal strategy’ for areas with high seasonal malaria transmission – aiming towards balancing factors affecting vaccine coverage and duration of protection. This would comprise administering doses 1–3 of RTS,S through routine EPI (with slight variability in scheduling depending on country), whilst delivering or combining dose 4 through a campaign approach, e.g. with SMC directly or using SMC as means to facilitate vaccine referrals.

Consideration of different implementation strategies, particularly a hybrid model, was approached by dividing country participants into three groups. The following pros and cons for combining or integrating RTS,S roll-out according to malaria seasonality and together with SMC schedules were collated:

Possible research questions in the context of malaria vaccine administration in conjunction with SMC were raised through the group work, these are listed in additional file 1.

## Day 3 implementation research needs and evaluation strategies to document the implementation of RTS, S in terms of effectiveness, acceptability, feasibility, safety and coverage

*New vaccine Post-introduction Evaluation (PIE) as tool* (Speakers: Jenny Walldorf, WHO, Geneva, Switzerland; Naziru Tanko Mohamed, EPI, Ghana).

The scope of using the PIE [10] as tool in the context of the introduction of new vaccines was provided in the presentation from WHO, with an overview of its objectives, methods, and specific considerations for malaria vaccines. The methods covered the whole process from areas of evaluation, preparatory steps and timelines, data collection tools, site selection, data analysis aspects, through to debrief, report and follow-up aspects. It was highlighted that PIE questionnaires have been adapted for malaria vaccine and used by the three pilot countries, Kenya, Malawi and Ghana. PIEs are not a prerequisite for the introduction of each vaccine; however, they are strongly encouraged for early malaria vaccine introducing countries to evaluate new delivery strategies and potential programmatic challenges (new visits, subnational implementation, seasonal delivery). A caveat is that a PIE conducted 6–12 months after introduction will not allow evaluation issues related to delivery of the 4th dose, therefore a second PIE or more targeted evaluation may be required at a later stage of the in implementation.

**Fig. 3:**
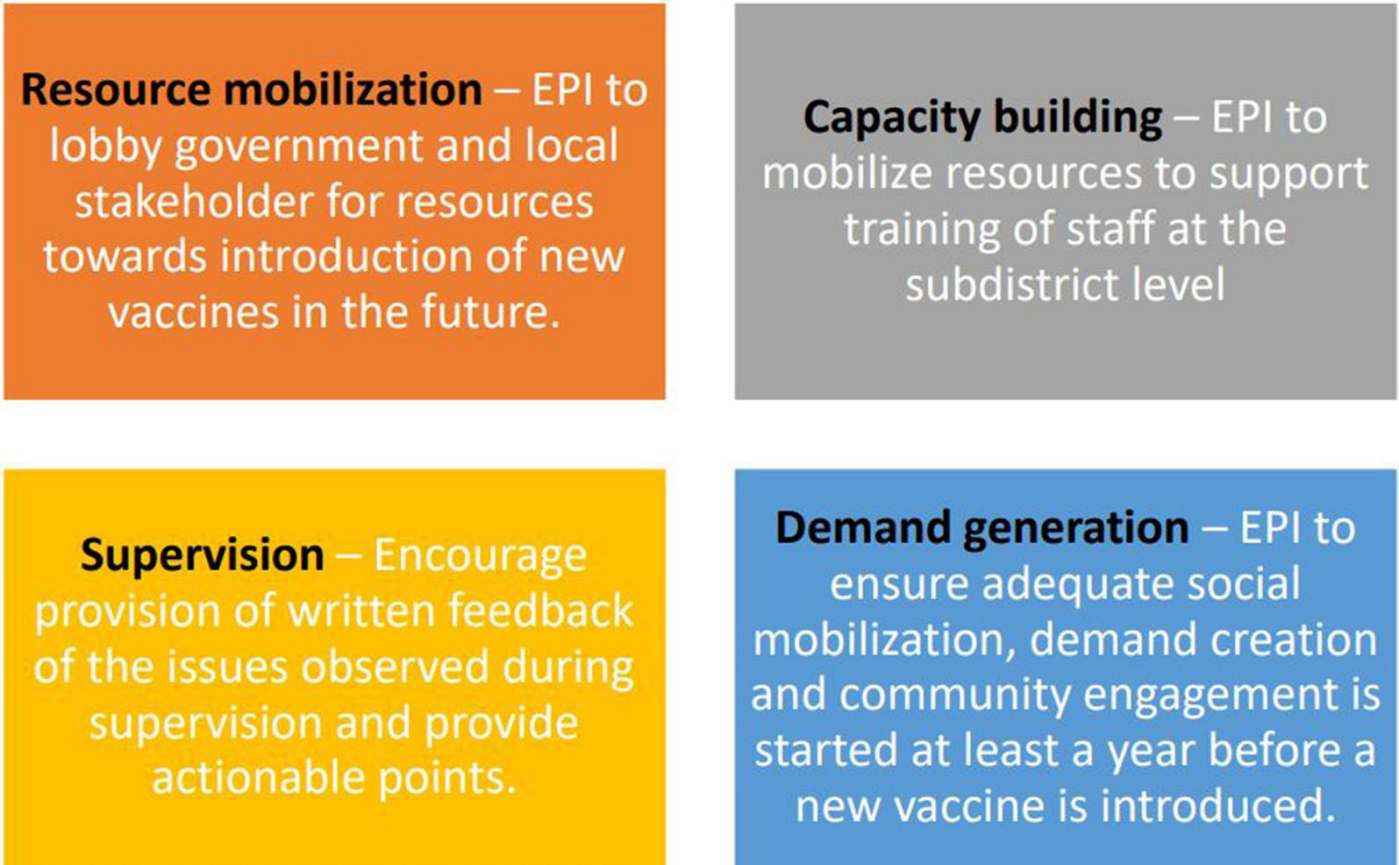
Recommendations from Post-introduction Evaluation (PIE) conducted across RTS,S pilot countries (Ghana, Malaria and Kenya)

As an exemplar, the experiences with PIE in the context of RTS,S piloting in Ghana were provided by Naziru Mohammed Tanko. The PIE was conducted using an adapted PIE tool with a mixed method approach to assess pre-implementation planning and vaccine introduction; social mobilization, advocacy and community engagement; healthcare worker training, understanding and application of eligibility criteria; logistics and resources for key MVIP activities; data collection, management and use; and vaccine safety surveillance. This allowed to bring out a number of strengths and areas for improvement in the vaccine implementation approach. The resulting recommendations centered around resource mobilization, capacity building, supervision and demand generation (see Fig. 3). The overarching conclusions from the PIE exercise indicated that: i) The RTS,S malaria vaccine introduction was generally successful; ii) Immunization services were improved and the collaboration between EPI and other programmes strengthened, particularly the National Malaria Elimination Programme (NMEP); iii) despite some misinformation propagated through social media in the early phase of vaccine introduction, coverage increased steadily – an indication of improved public confidence in the vaccine; and iv) applying learnings from the implementation is vital for successful wider roll-out (as well as the introduction of other new vaccines in the future).

*Case control studies to evaluate vaccine strategy efficacy* (Speakers: Thomas Gyan and Kwaku Poku Asante, Kintampo Health Research Centre, Kintampo, Ghana).

Colleagues from Ghana presented on behalf of the Malaria Vaccine Pilot Evaluation (MVPE-CC) their approach to apply an embedded case–control study as evaluation tool for Malaria Elimination Programmes introducing the RTS,S vaccine (or other malaria vaccines). This approach was employed specifically to address the following questions across Ghana, Kenya and Malawi: (1) Are children who receive RTS,S vaccination at increased risk of meningitis compared to unvaccinated children? (2) Are children who receive RTS,S vaccine at increased risk of cerebral malaria compared to unvaccinated children? (3) Is the incidence of severe malaria increased in children who received 3 doses, but failed to receive a 4th dose, compared to children who did not receive the vaccine (the rebound effect)? (4) What is the effectiveness of RTS,S (following 3 doses, and following the 4th dose) in preventing severe malaria? and (5) Is there any evidence that RTS,S vaccine increases mortality in girls, or is less effective in preventing death in girls, compared to boys? The pilot evaluations have shown that the vaccine is safe in routine use, but was unable to show the added benefit of a 4th dose (question 4) and whether a 3-dose schedule is a cost-effective option in some situations. The added individual-level evidence on safety will provide added reassurance on the vaccine safety.

Details of study design including governance structure, case definitions, data collection and analyses approaches, as well as ethical considerations were laid out, with provision of limitations and challenges. To date the study recruited over 1500 cases and 6000 controls. Whilst the work is ongoing, the team are preparing a manual for the case control approach, a budget template to help estimate the cost of such an evaluation, webinars to disseminate the findings when available as well as a publication.

*Implementation questions and funding for evaluation and implementation research questions* (Speakers: Mary Hamel, Malaria Vaccines, WHO, Geneva, Switzerland and Stephen Sosler, Gavi, Geneva, Switzerland).

The pilot introduction of RTS,S in Malawi, Kenya and Ghana have provided many rich lessons on safety in routine use, acceptability, feasibility, impact, and the use of remote tools for effective delivery during a pandemic. Nonetheless, outstanding questions remain on seasonal vaccine delivery; programmatic optimization of delivery in different settings; increasing supply and reducing cost; optimizing impact, including combined use of vaccine and other malaria prevention measures; as well as behaviour change and communication, including optimal use of additional visits to catch up on other vaccines and child health measures.

Notably, the pilots were conducted in areas with functional EPI and NMCP programmes, in areas with perennial transmission, and the vaccines were provided through routine EPI (age-based administration throughout the year). As discussed over the course of the workshop, the initial subnational introduction of the vaccine in highest need (category 1) areas may entail delivery through weaker health systems and areas with high malaria seasonality may select a seasonal delivery, thereby warranting a broader spectrum of implementation research questions. This could further be expanded to include research on vaccine escape mutant, immunogenicity, co-administration studies.

The WHO and Gavi are engaging PMI Insights jointly with a research or public health institution in a malaria-affected country, to develop a comprehensive and vetted research agenda. A broad and inclusive stakeholder consultation process will be undertaken to gather input from ministries of health, research institutions and partners engaged in malaria vaccine introduction or research, CSO, regional and global bodies, and global health funding agencies. The aim is to conduct a rigorous evaluation process to prioritize and rank research topics and develop research agenda.

### General discussion on the research agenda, potential additional implementation research questions to be considered and funding opportunities

The final discussion session focused on areas that needed additional clarification (for instance regarding Gavi application process) and further exploration of research questions. These discussion strands have been incorporated into the overall report including the additional files.

## Conclusions and outcomes

The overall conclusions from the workshop are summarised below, highlighting more specific areas relating to supply and demand as well as lessons learned to date. Further, open questions and suggested topics for future research were collated (see Additional file 1), and proposed steps going forward are included in the below.

This workshop was well received by participants. The experiences and lessons learned in the three pilot countries were valuable to inform planning for vaccine roll-out in other countries. A number of scientific and programmatic questions remain regarding the implementation of malaria vaccines especially in areas with poor EPI or health system performance, and where seasonal delivery strategies may be needed. Countries are considering approaches they may use. It will be important that introduction of malaria vaccines in early implementing countries are carefully evaluated to find out what works best.

### Supply and demand

- There is great demand for a malaria vaccine, coupled with high acceptability of RTS,S to date. This has translated into an unprecedented demand with 13 applications submitted to Gavi at the first opportunity in early 2023.
- The initial supply for RTS,S cannot meet this demand. Countries therefore may need to take difficult decisions to prioritize areas where the vaccine will be introduced initially.
- Beyond the three pilot countries (Ghana, Kenya, Malawi), it is unlikely that countries will introduce RTS,S before beginning of 2024.

### Lessons learned to date from the pilot countries and other research studies

- Awareness of differences in RTS,S vaccine efficacy depending on epidemiological context and timing of vaccination in relation to the malaria transmission season can support informed decision-making by EPI and NMCP regarding the optimal implementation strategy. Training of personnel administering the vaccine should be carefully timed prior to the introduction of new vaccines, though not too far ahead.
- Community engagement from the outset is key to increase acceptability of the vaccine where it is introduced.
- Dialogue and integration of activities by key national stakeholders, e.g. the EPI and NMCP, is mutually beneficial, as evidenced in the RTS,S pilot countries. For countries with weaker EPI performance and/or where malaria transmission is highly seasonal, synergies between the two programmes through joint-up of implementation strategies has the potential to improve uptake of all available interventions.
- Achieving high coverage of dose 4 has been challenging, even under favourable conditions in pilot countries where EPI coverage overall is good. This suggests the need for additional efforts, or alternative approaches to cover the harder-to-reach and strengthened approaches to increase vaccine coverage during the second year of life.
- Optimal impact of RTS,S is achieved with four doses and – where applicable – by timing of vaccine administration prior to higher malaria transmission periods in highly seasonal areas [4].
- Findings from the pilot countries are extremely valuable towards informing the introduction of RTS,S in other settings.

### Open questions and research agenda

- The workshop helped to collate an initial set of questions and more general areas for future investigation (Additional file 1) for further consideration and exploration in future. It was acknowledged that many implementation research questions remain and will continue to arise as and when countries begin implementation.

### Possible steps moving forward

- In the context of seasonal malaria, an ‘optimal RTS,S schedule’ resulting in maximum vaccine efficacy balanced against cost-effectiveness and feasibility, may require a hybrid delivery model with administration of doses 1–3 based on a child’s age through routine EPI (with slight variability in scheduling depending on country), and delivery of dose 4 through a campaign (or similar) approach before or at the onset of the rainy season. Fourth dose delivery could be coordinated with preparations for SMC delivery (or other mass drug administration programmes), e.g. advanced communications for SMC or using SMC as means to facilitate vaccine referrals. However, there is a great need for exploration of the feasibility, acceptability and cost effectiveness of this model through implementation research.
- Modelling approaches could be explored to provide guidance regarding this hybrid model in meantime particularly with respect to uptake of the vaccine in the 2nd year of life (4th dose) and optimal spacing between the 3rd and 4th dose of RTS,S.
- As recommended by the WHO, countries should document lessons learned from seasonal vaccination strategies, especially regarding operational feasibility, vaccine efficacy and safety. The ADP is considering provision of support for countries to develop such documentation.
- Continued information exchange between countries planning to introduce RTS,S should be facilitated, e.g. through future workshops. Further, suitable communication materials should be developed (technical reports may not be accessible) for the community of implementers from vaccine and NMCP programmes.
- For the development of a comprehensive and vetted research agenda (facilitated by the WHO and Gavi) consultation with researchers and other stakeholders on the ground is essential, to capture relevant implementation priorities generated in the global south. This is planned for later in 2023.

### Supplementary Information

**Additional file 1.** Exemplar research questions and areas for investigation in the context of implementing a malaria vaccine and in settings with seasonal malaria transmission.

**Additional file 2.** TDR workshop website including workshop presentations. A dedicated TDR website was set up in conjunction with this workshop, this includes a short report in English and French, as well as links to the workshop presentations and other relevant information.

**Additional file 3.** Workshop agenda: Implementation strategies for the introduction of the RTS,S/AS01 (RTS,S) malaria vaccine in countries with areas of highly seasonal transmission.

## Data Availability

Not applicable.

## Abbreviations

ADP: Access and Delivery Partnership
Gavi: Gavi, the Vaccine Alliance
LLINs: Long-lasting insecticidal nets (LLINs)
LMIC: Low- and middle-income country
LSHTM: London School of Hygiene & Tropical Medicine
OPT-SMC: Optimizing seasonal malaria chemoprevention project
PIE: Post-introduction evaluation
SMC: Seasonal malaria chemoprophylaxis
TDR: Special Programme for Research & Training in Tropical Diseases (TDR)
WHO: World Health Organization
WHO MVIP: WHO Malaria Vaccine Implementation Programme
