# Supplementary material for: Implementation strategies for the introduction of the RTS,S/AS01 (RTS,S) malaria vaccine in countries with areas of highly seasonal transmission: workshop meeting report
Source: Malar J. 2023 Aug 23;22:242. doi: 10.1186/s12936-023-04657-5 (PMC10464391; doi:10.1186/s12936-023-04657-5)
Supplement: Supplementary file 1 — Additional file 1. Exemplar research questions and areas for investigation in the context of implementing a malaria vaccine and in settings with seasonal malaria transmission. [file 12936_2023_4657_MOESM1_ESM.docx]

# Supplementary information

## Additional file 1: Exemplar research questions and areas for investigation in the context of implementing a malaria vaccine and in settings with seasonal malaria transmission

The primary implementation research question in the context of RTS,S in countries with varying degrees of EPI coverage and seasonality remains: What is the optimal schedule for the delivery of the RTS,S vaccine to achieve highest impact (efficacy) whilst maintaining operational feasibility?

Specific research areas and questions raised during the workshop were collated as follows:

**Community engagement and acceptability**

- How can vaccine misconceptions be addressed in different settings?
- When and how should engagement with community leaders take place?
- What means could be employed to increase acceptability of RTS,S in areas where SMC is routinely delivered (engagement of health workers, caregivers, wider community)?
- Should RTS,S be subject to parental consent or be considered as for all other routine vaccinations?

**Optimisation and feasibility of RTS,S delivery in SMC settings with variable EPI coverage**

- Optimizing delivery of child health interventions through integration of services or programmes
  - Can delivery of the RTS,S dose 4 and/or SMC help increase uptake of other child health interventions such as other vaccines, ITN use, Vitamin A, deworming etc. and vice versa?
  - What are the possible assess strategies to reach those with zero EPI doses and under-served populations leveraging community demand for malaria vaccine?
- Piloting models of combined or linked vaccine and SMC delivery through
  - Co-administration via one team covering interventions
  - Parallel administration via EPI and NMCP teams
  - Sequential administration, e.g. through referrals by SMC teams to EPI teams
- What is the cost effectiveness of combined vaccine and SMC delivery models?
- Areas to evaluate in terms of operational feasibility for co-administration
  - logistics
  - programming AA
  - adverse drug event monitoring
- Evaluation of optimal target age group for a campaign-style delivery of dose 4
  - Context of limited RTS,S supply in the initial phase of the vaccine roll-out
- What is the most feasible delivery strategy given malaria transmission intensity and length of the transmission season?

**Vaccine scheduling, efficacy & safety**

- What is the level and duration of vaccine efficacy following the 4^th^ dose of RTS,S (vis-à-vis the possible need for subsequent annual doses)?
- What is the optimal age range for vaccination efficacy (consideration of vaccine efficacy in younger children <5 months and older children > 17 months)?
- What is the best schedule to maximise efficacy depending on malaria transmission intensity and length of the transmission season?
- Could fractional dosing be considered vis-à-vis vaccine efficacy and dose sparing?
- What are the potential benefits of clearing malaria parasitaemia prior to vaccination to increase vaccine efficacy?
- With introduction of RTS,S, can SMC be reduced over time?
- What is the optimal interval in terms of efficacy and safety between dose 3 and 4 (modelling studies might be informative)?
- Do additional annual doses need to be considered after the initial vaccine course (i.e safety and efficacy of >4 doses)?
- Is there a need for a ‘coverage target’ for vaccination campaigns (context: No herd immunity with the malaria vaccine)?
- How can vaccine effectiveness and safety be monitored, where RTS,S may be introduced seasonally (in view of possible adaptation of schedule)?
- What is the safety of co-administration of RTS,S and SMC through a campaign strategy?
- Are there added/synergistic effects of the malaria vaccine with Perennial Malaria Chemoprevention (PMC)?

**Monitoring & evaluation; Digital technology**

- Post-introduction Evaluation (PIE) – methods for evaluation of RTS,S delivery in SMC settings
- Implementation research on opportunities for digitalisation of vaccination / adverse drug event monitoring

**General**

- Documentation and sharing of best practices from (sub-)national implementation of RTS,S (e.g. in high priority areas)
- Interchangeability of RTS,S and candidate malaria vaccine R21
