# Supplementary material for: Implementation strategies for the introduction of the RTS,S/AS01 (RTS,S) malaria vaccine in countries with areas of highly seasonal transmission: workshop meeting report
Source: Malar J. 2023 Aug 23;22:242. doi: 10.1186/s12936-023-04657-5 (PMC10464391; doi:10.1186/s12936-023-04657-5)
Supplement: Supplementary file 2 — Additional file 2. TDR workshop website including workshop presentations. A dedicated TDR website was set up in conjunction with this workshop, this includes a short report in English and French, as well as links to the workshop presentations and other relevant information. [file 12936_2023_4657_MOESM2_ESM.docx]

# Supplementary information

## Additional file 2: TDR workshop website including workshop presentations

A [dedicated TDR website was set up in conjunction with this workshop](https://tdr.who.int/activities/implementation-strategies-for-delivering-the-rts-s-as01-malaria-vaccine-in-countries-with-seasonal-transmission), this includes a short report in English and French, as well as links to the workshop presentations and other relevant information.
