# Supplementary material for: Implementation strategies for the introduction of the RTS,S/AS01 (RTS,S) malaria vaccine in countries with areas of highly seasonal transmission: workshop meeting report
Source: Malar J. 2023 Aug 23;22:242. doi: 10.1186/s12936-023-04657-5 (PMC10464391; doi:10.1186/s12936-023-04657-5)
Supplement: Supplementary file 3 — Additional file 3. Workshop agenda: Implementation strategies for the introduction of the RTS,S/AS01 (RTS,S) malaria vaccine in countries with areas of highly seasonal transmission. [file 12936_2023_4657_MOESM3_ESM.docx]

# Supplementary information

## Additional file 3: Workshop agenda: Implementation strategies for the introduction of the RTS,S/AS01 (RTS,S) malaria vaccine in countries with areas of highly seasonal transmission

*Note: The agenda was slightly adapted during the meeting to accommodate discussion time and technical issues.*

| **Time (GMT)** | **Topic** | **Speakers** |
| --- | --- | --- |
| **Day 1 Monday 23^rd^ January 2023**  **Setting the scene** | | |
| **Chairperson: Dr Mahamat Saleh Issakha Diar** | | |
| 9:00-9:10 | Welcome and opening of the meeting | Chancellor of university of Thiès  Prof Ramatoulaye Diagne Mbengue |
| 9:10-9:20 | Meeting objectives and introduction of participants | Jean Louis Ndiaye (Univ Thiés) &  Corinne Merle (TDR) |
| 9:20-9:40 | Introduction to OPT-SMC (5min)  Introduction to ADP (5min)  Q&A (10min) | Jean Louis Ndiaye (Univ Thiés)  Cecilia Oh (UNDP, remote) |
| 9:40-10:10 | RTS, S malaria vaccine current evidence, including efficacy, safety, feasibility and impact and update on R21 (20+10 min Q&A) | Mary Hamel (WHO MVIP, IVB, remote) |
| 10:10-10:25 | Vaccine efficacy and seasonality of malaria (10+5min Q&A) | Paul Milligan (LSHTM) |
| 10:25-10:40 | Update on supply of RTS,S with allocation framework (10+5min Q&A) | Eliane Furrer (WHO MVIP, remote) |
| 10:40-10:55 | Gavi malaria vaccine programme update (10+5min Q&A) | Stephen Sosler (Gavi, remote) |
| ***10:55-11:30*** | ***Break*** | |
| 11:30-11:50 | Practical considerations for RTS, S malaria vaccine supply chain and limited supply management at country level (10+10min Q&A) | Betsy Wilskie (PATH) |
| 11:50-12:00 | Regulatory consideration for introduction of RTS, S in countries with seasonal malaria (10+10min Q&A) | Lydia Tuitai (WHO AFRO) |
| 12:00-13:00 | Round table on plans for RTS, S roll-out in countries who applied in January 2023 for RTS, S vaccine procurement | Countries |
| ***13:00-14:30*** | ***Lunch*** | |
| **Chairperson: Dr Olimatou Kolley** | | |
| 14:30-14:45 | Overview of SMC (target population, eligible geographic area, model of delivery, integration with other preventive measures, etc.) (10+5min Q&A) | André Tchouatieu (MMV) |
| 14:45-15:15 | Lessons learned and practical experience from pilot introduction of the RTS, S malaria vaccine: implementation in routine child immunization programmes and possible implications for implementation through SMC or other mass drug administration programmes  Kenya (10min)  Malawi (10min)  Ghana (10min) | Rose JALANG’O (EPI, Kenya)  John Sande (EPI, Malawi) and Brenda Lupafya MHONE (NMCP)  Mohamed Naziru Tanko (EPI, Ghana) and Muniratu VENU (NMCP, Ghana) |
| 15:15-15:30 | Qualitative findings and lessons learned from the pilot countries: perceptions of malaria, the vaccine and other interventions | Scott Gordon (PATH) |
| 15:30-16:00 | Discussion on country lessons learned and qualitative findings | All |
| ***16:00-16:30*** | ***Break*** | |
| 16:30-16:45 | Considerations and possible modalities for introduction of RTS,S malaria vaccine in countries with seasonal malaria | Rafiq Okine (WHO MVIP, remote) |
| 16:45-17:15 | Brainstorming - collating questions to be addressed in break-out groups on day 2 under different modalities of RTS,S malaria vaccine introduction in countries with seasonal malaria. | All |
| 17:15-17:30 | Wrap-up of Day 1 | |
| ***18:30*** | ***Dinner*** | ***Venue to be confirmed*** |
| **Day 2 Tuesday 24th January 2023**  **Implementation strategies and mode of delivery for combined RTS, S and SMC programmes**  **Parallel sessions** | | |
| 9:00-13:00 | Groupwork on implementation strategies depending of model delivery defined on day 1  (Break out groups) | All |
| ***13:00-14:30*** | ***Lunch*** | |
| **Chairperson: Dr Marcellin Ateba** | | |
| 14:30- 17:00 | Feedback from breakout groups & discussion | All |
| 17:00-17:15 | Wrap-up of Day 2 | |
| **Day 3 Wednesday 25th January 2023**  **Implementation research needs and evaluation strategies to document the implementation of RTS, S in terms of effectiveness, acceptability, feasibility, safety and coverage** | | |
| **Chairperson: Scott Gordon** | | |
| 9:00-9:45 | New vaccine post-introduction Evaluation (PIE) as tool [WHO_IVB_10.03_eng.pdf](https://apps.who.int/iris/bitstream/handle/10665/70436/WHO_IVB_10.03_eng.pdf?sequence=1&isAllowed=y) (20 min presentation & 25 min discussion) | Jenny Walldorf (WHO MVIP, remote) and Mohamed Naziru Tanko |
| 9:45 – 10:30 | Case Control studies to evaluate vaccine strategy efficacy | Thomas Gyan & Kwaku Poku Asante (Kintampo Health Research Centre, Ghana) |
| ***10:30-11:00*** | ***Break*** | |
| 11:00-11:15 | Implementation questions and funding for evaluation and implementation research questions (15min) | Stephen Sosler (Gavi, remote) & Mary Hamel (WHO MVIP, IVB, remote) |
| 11:15–12:30 | General discussion on the research agenda, potential additional implementation research questions to be considered and funding opportunities | All |
| 12:30-13:15 | Overall conclusions & next steps | All |
| ***13:15-14:30*** | ***Lunch*** | |
| **END OF MEETING** | | |
